# Influence of high-energy laser therapy to the patellar tendon on its ligamentous microcirculation: An experimental intervention study

**DOI:** 10.1371/journal.pone.0275883

**Published:** 2023-03-27

**Authors:** Andreas Brandl, Christoph Egner, Ursel Reisser, Christian Lingenfelder, Robert Schleip

**Affiliations:** 1 Faculty for Psychology and Human Movement Science, Department of Sports Medicine, Institute for Human Movement Science, University of Hamburg, Hamburg, Germany; 2 DIPLOMA Hochschule, Bad Sooden-Allendorf, Germany; 3 Zimmer Medizinsysteme, Neu-Ulm, Germany; 4 Department of Sport and Health Sciences, Conservative and Rehabilitative Orthopedics, Technical University of Munich, Munich, Germany; Uniklinik RWTH Aachen: Universitatsklinikum Aachen, GERMANY

## Abstract

Laser therapeutic applications, such as the use of high energy lasers (HILT), are widely used in physical therapy, but basic studies on the mechanisms of action of HILT on tendinous/ligamentous tissue are largely lacking. The aim of this study was to investigate microcirculatory changes of the patellar tendon by HILT. 21 healthy volunteers were included in the present investigation. Before and after HILT, as well as 10 minutes later, the microcirculation was measured by noninvasive laser Doppler and white light spectroscopy (O2C device). Tissue temperature was recorded at the measurement time points using thermography. Blood flow increased significantly by 86.38 arbitrary units (AU; p < 0.001) after the intervention and by 25.76 AU (p < 0.001) at follow-up. Oxygen saturation increased by 20.14% (p < 0.001) and 13.48%, respectively (p < 0.001), whereas relative hemoglobin decreased by 6.67 AU and 7.90 AU, respectively. Tendon temperature increased by 9.45° and 1.94° Celsius, respectively. Acceleration of blood flow by improving the flow properties of erythrocytes and platelets may have caused the results. HILT could be a therapeutic perspective for tendon pathologies with impaired microcirculation, although further studies are needed to validate the experimental results.

## Introduction

Laser therapy applications are widely used in physical therapy [1–3]. However, there is a lack of empirical research on fascial structures, their variability and adaptability [4]. Non-invasive therapy devices are becoming increasingly important in the context of musculoskeletal rehabilitation [5,6]. In contrast to low-energy laser therapy of class 3B with a power of less than 500 mW, high-intensity laser therapy (HILT) typically uses class 4 laser diodes that deliver light with a power of at least 500 mW [7]. Although HILT was described as a method for treating myofascial tissue (e.g., knee osteoarthritis or epicondylitis) [1], basic studies on the mechanisms of action of HILT on dense parallel-fibred connective tissues (such as tendons or ligaments) are scarce [8].

Vascular factors were identified in previous studies as critical for the development of tendinopathies [8,9]. Reduced blood flow, especially after activity, represented the greatest risk factor [9]. Studies in the Achilles tendon showed that structural parameters—such as Achilles tendon thickness, tendon structure as analyzed by ultrasound analysis, or foot posture index–did not significantly contribute to the prediction of Achilles tendinopathy [8,10], whereas microcirculation in the capillary venous area served as a significant risk predictor [8].

Furthermore, it is observed, that microcirculation in tendon tissue plays a prominent role in regeneration after injury. In this context, oxygen, blood viscosity, endothelial functionality and blood vessel permeability are important parameters that emphatically influence wound healing [11].

Hypoxia in tendon tissue promotes the production of proinflammatory cytokines, which can be observed in the early stage of the development of tendinopathy and is therefore discussed as a trigger for such a condition. These hypoxia-related proteins are key mediators of cellular inflammation and apoptosis, processes that cause a shift in collagen matrix synthesis toward an increased proportion of type III collagen (which tends to form fibrils with lower mechanical strength). A shift in matrix structure from type I collagen, which makes up 70% of the dry mass in healthy tendon tissue, to type III collagen reduces the resistance of the tendon to tensile forces, thereby favoring ruptures [9]. Hypoxia further causes local acidosis, corresponding to increased intratendinous lactate accumulation, which further negatively influences pathogenesis [12].

A photospectroscopic device for monitoring microcirculation in tendons and other tissues is the O2C (Oxygen to See: LEA Medizintechnik, Giessen, Germany), which combines white light and laser Doppler for real-time in vivo measurement of blood flow, oxygen saturation (SO_2_), and relative hemoglobin (rHb) [13]. The O2C has been used in a variety of disciplines [13], including myofascial and tendinous tissue [8,10,14]. Since it is likely that a change in the microcirculation of the superficial tendons also involves a change in skin temperature [8,10], infrared thermography has become a popular method for measuring such probable relationships [15]. This involves the generation of thermal images (thermograms) based on the infrared radiation emitted by objects, which allow safe, non-contact and non-invasive quantification of skin temperature [15].

Recent studies indicate that tendinopathies are associated with new vascularization with swelling and pain [16,17]. However, what seems like a paradox could, according to Järvinen [17], also represent persistent preceding hypoxia. Since tissue regeneration requires an adequate supply of oxygen and nutrients, therefore, neovascularization in tendinopathy can be interpreted as a sign of both persistent hypoxia and a failed attempt to repair the tendon [17]. It is known from the fields of cancer research and retinopathy that hypoxia-induced neovessels are hyper-permeable, resulting in vascular leakage and reduced blood flow. This provides insufficient oxygen and nutrients needed for tissue maintenance and possible regeneration [17].

HILT decreases erythrocyte deformability and platelet aggregation, resulting in membrane revitalization, viscosity reduction, and erythrocyte stress adaptation. In addition, the lifespan of erythrocytes and platelets is prolonged and their flow properties are improved under HILT [2,18,19]. Therefore, HILT is also discussed as a promising method for the treatment of various blood diseases [2,3,18–20].

The aim of this study was to investigate microcirculatory changes of the patellar tendon by HILT. The first hypothesis investigated in this study was that patellar tendon microcirculation will increase significantly after HILT. A second hypothesis was that this increase in blood flow will also significantly increase skin temperature.

## Methods

### Study design

**Fig 1:**
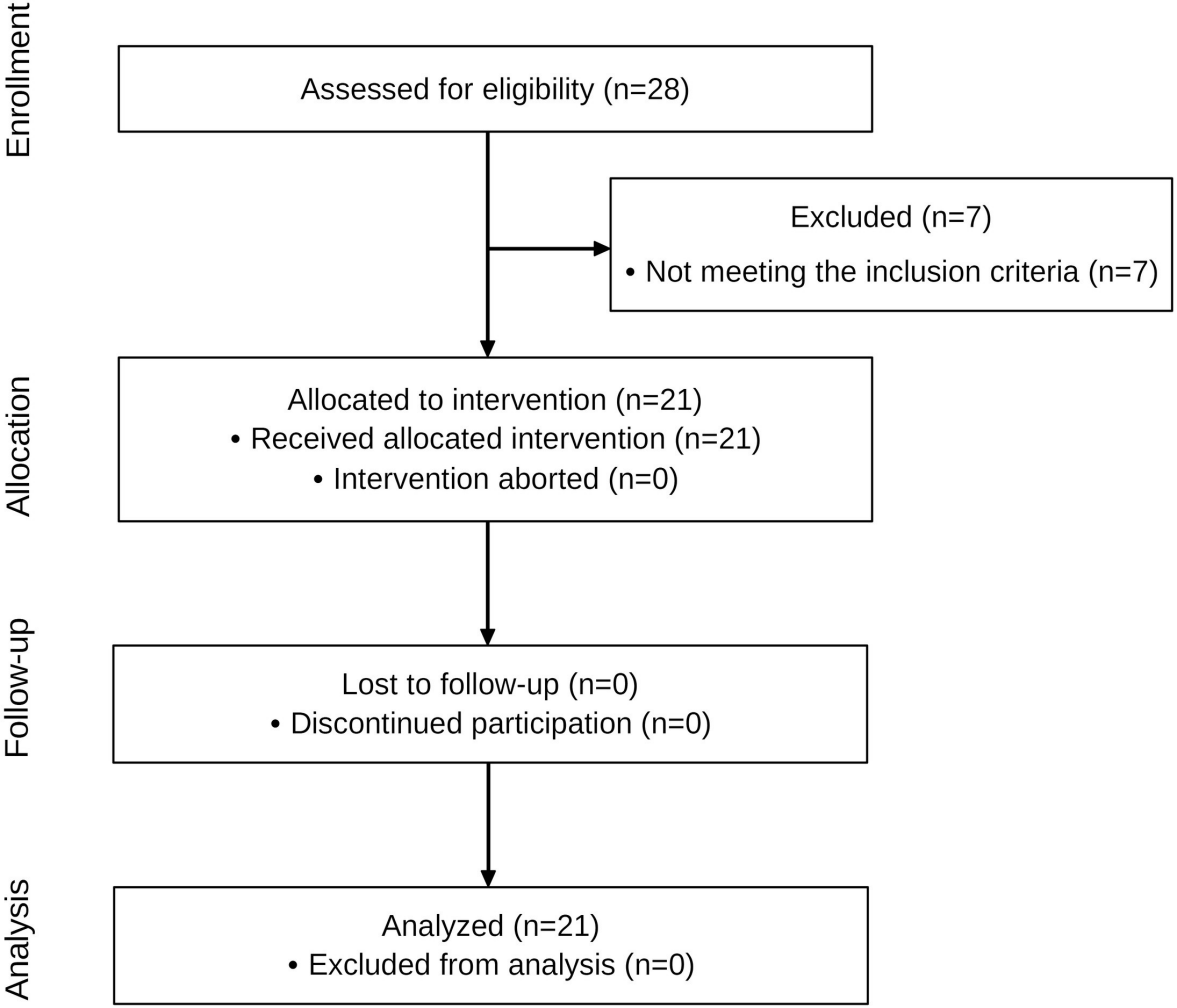
Flow diagram of the study.

The experimental intervention study was conducted prospectively with a group receiving HILT. Measurements were taken before and after the HILT application and at a 10-minute follow-up according to the STROBE guidelines (Fig 1) [21]. The study protocol was prospectively registered with the German Clinical Trials Register (DRKS00028155) on 18.02.2022. The study has been reviewed and approved by the ethical committee of the DIPLOMA Hochschule, Germany (Nr. 1021/2021), has been carried out in accordance with the declaration of Helsinki and has obtained written informed consent from the participants [22].

**Fig 2:**
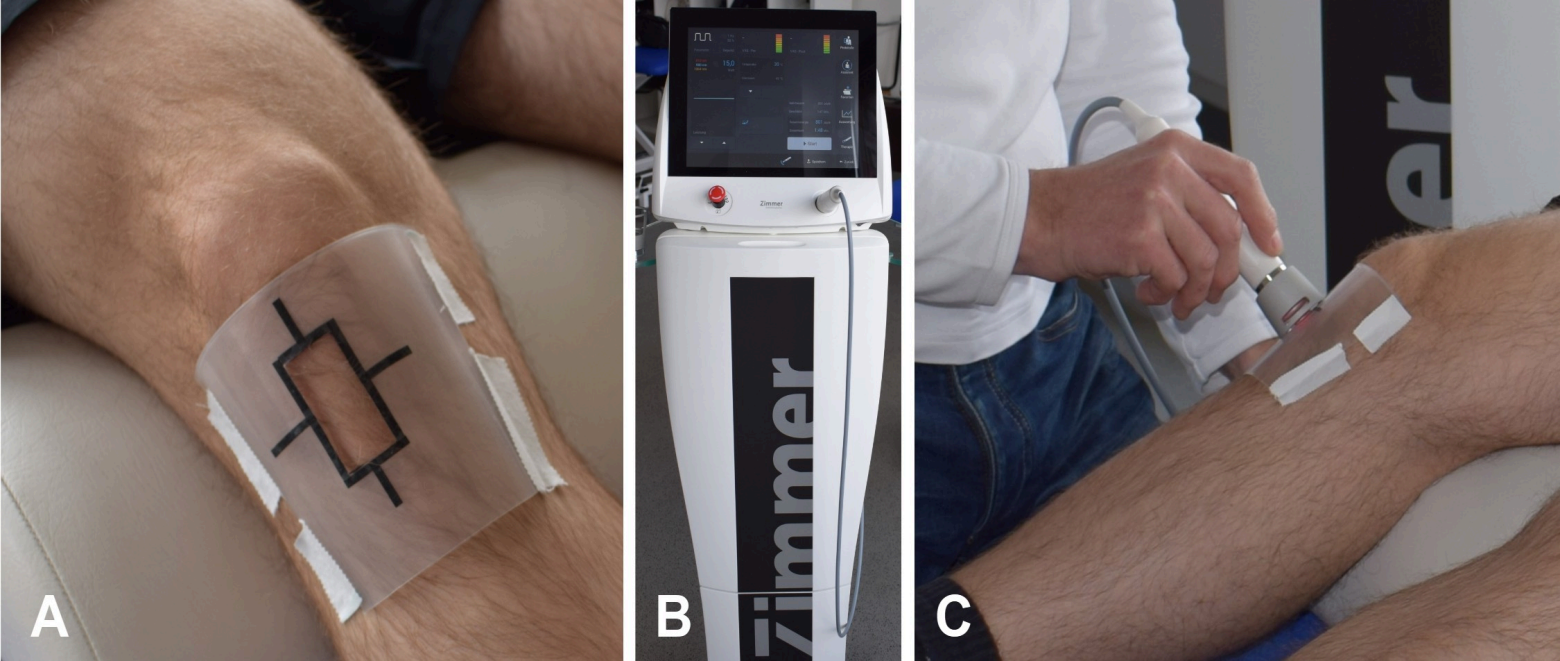
A show the prefabricated template defining the treatment area of 2 cm x 4 cm. B shows the OptonPro high intensity laser therapy device. C shows the laser application to the patellar tendon.

First, the anthropometric data, age, gender, height, and weight were collected by the investigators (AB, RS). Before the measurements, the participants were given information regarding the implementation of the intervention and measurements. They lay supine with their heads bent at 160° (cervical spine flexion) on a treatment table. The knees were placed underneath and a knee flexion of 145° - 150° was preset. The measurements were taken in daylight between 9:00 am and 03:00 pm and at a room temperature of 22 degrees Celsius. The initial measurement (baseline) was performed first. Then the HILT application (OptonPro, Zimmer Medizinsysteme GmbH, Neu-Ulm, Germany, Fig 2B) was carried out (Fig 2C). A total amount of energy of 800 J (100 J/cm2) was applied to a predefined area of 2 cm x 4 cm on the patellar tendon (Fig 2A). The post-measurement was performed immediately after the intervention. To distinguish solely a previously observed heat-dependent effect from putative laser-specific effects, a 10-minute follow-up measurement was carried out [23].

### Participants

The study was conducted in a school of physiotherapists, in a medium-sized city in middle Germany. The number of participants was calculated based on a previous examination of the results of a heat therapy study and was set at 18 (Cohen’s d = 1.2, α err = 0.05, 1-β err = 0.9) [23]. Assuming a maximum of three participants dropped out, we enrolled n = 21 participants. The acquisition was carried out via direct contact, a notice board, and the distribution of information material in the school.

Inclusion criteria were: (a) generally healthy constitution; (b) the persons to be treated must had to have an intact thermal sensitivity and be able to perceive and communicate pain; (c) BMI between 18 and 29.9; (d) female or male participants aged 18 to 60 years; (e) sitting position for 15 minutes had to be pain-free for the participants.

Exclusion criteria were: (a) generally valid contraindications to physiotherapeutic and osteopathic treatments of the patellar tendon (i.e., fractures, tumors, infections, severe cardiovascular and metabolic diseases); (b) pregnancy; (c) rheumatic diseases; (d) taking medication that affects blood circulation, pain or mind; (e) taking muscle relaxants; (f) skin changes (e.g. neurodermatitis, psoriasis, urticaria, decubitus ulcers, hematoma), (g) surgery or other scars in the patellar tendon region; (h) previous mental illness; (i) surgery in the last three months; (j) prosthetics or internal knee arthroplasty; (k) acute inflammation; (l) diseases affecting the microcirculation of the lower limbs (e.g. peripheral arterial occlusive disease, venous insufficiency).

### Blood flow measurement

**Fig 3:**
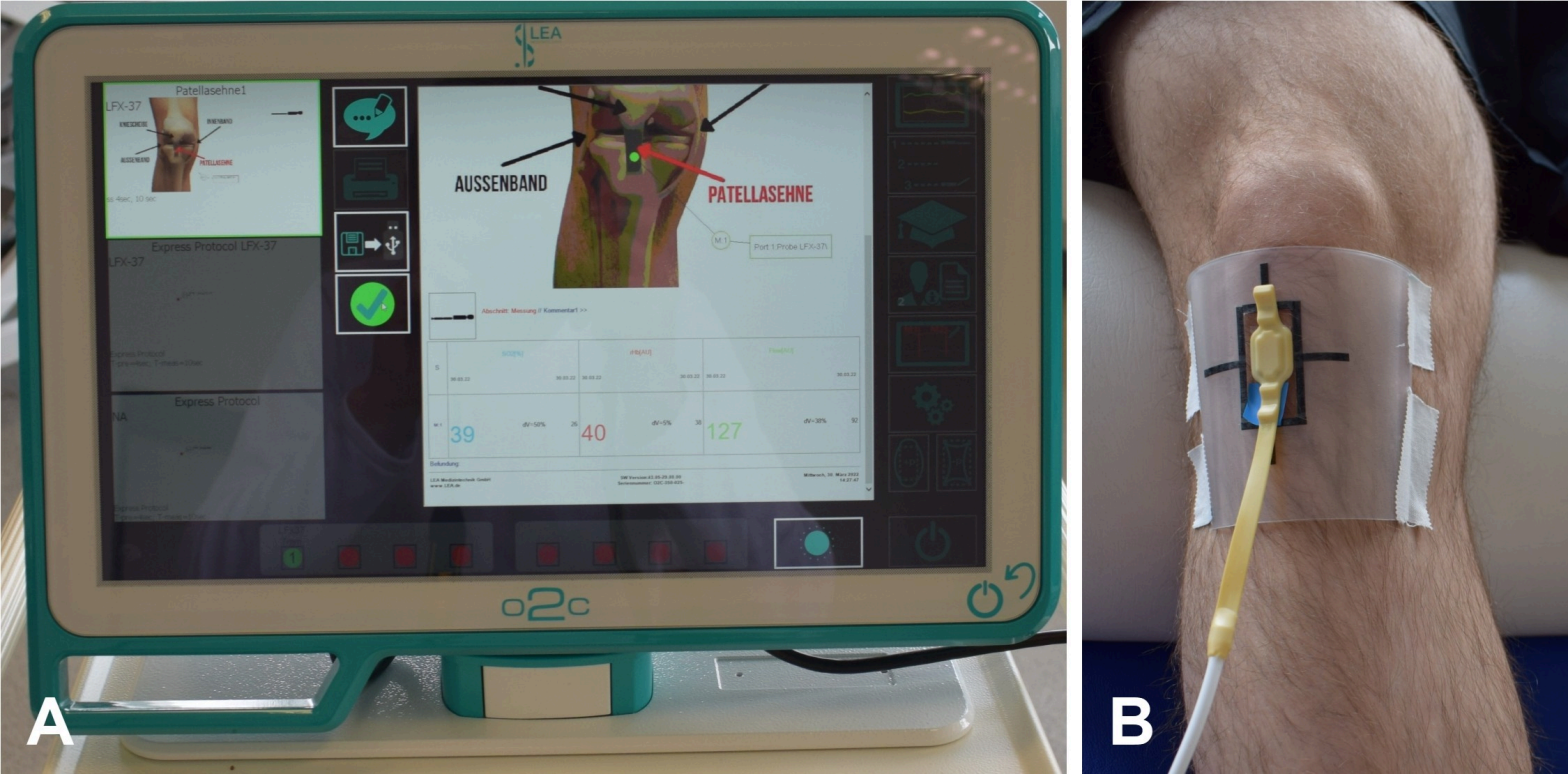
Hardware for microcirculation measurement using a laser Doppler flowmeter and a tissue spectrometer. B: Probe placement. A:

Measurement of the microcirculation of the patellar tendon was performed using a laser Doppler flowmeter and white light tissue spectrometer (O2C, LEA Medizintechnik GmbH, Heuchelheim, Germany) (Fig 3). This made it possible to simultaneously determine blood flow, SO_2_, and rHb of the measured tissue. The device determined these parameters at the venous end of the capillary and thus provided information about the local microcirculation. The measurement procedure is described in detail in Brandl et al. [14]. Briefly, the average values over 10 seconds were derived at a sampling rate of 40 Hz for blood flow by laser Doppler spectroscopy and 2 Hz for SO_2_ and rHb by white light spectroscopy. Environmental factors such as light and temperature (22° Celsius) were kept constant for standardization. An experienced researcher (AB) performed all measurements. The O2C fiber probe was fixed pressure-free with a transparent double-sided adhesive film (Lea Medizintechnik, Giessen, Germany). The ICC value for the measurement was calculated in a previous study and was reported to be excellent (ICC = 0.99) [14].

### Skin temperature measurement

**Fig 4:**
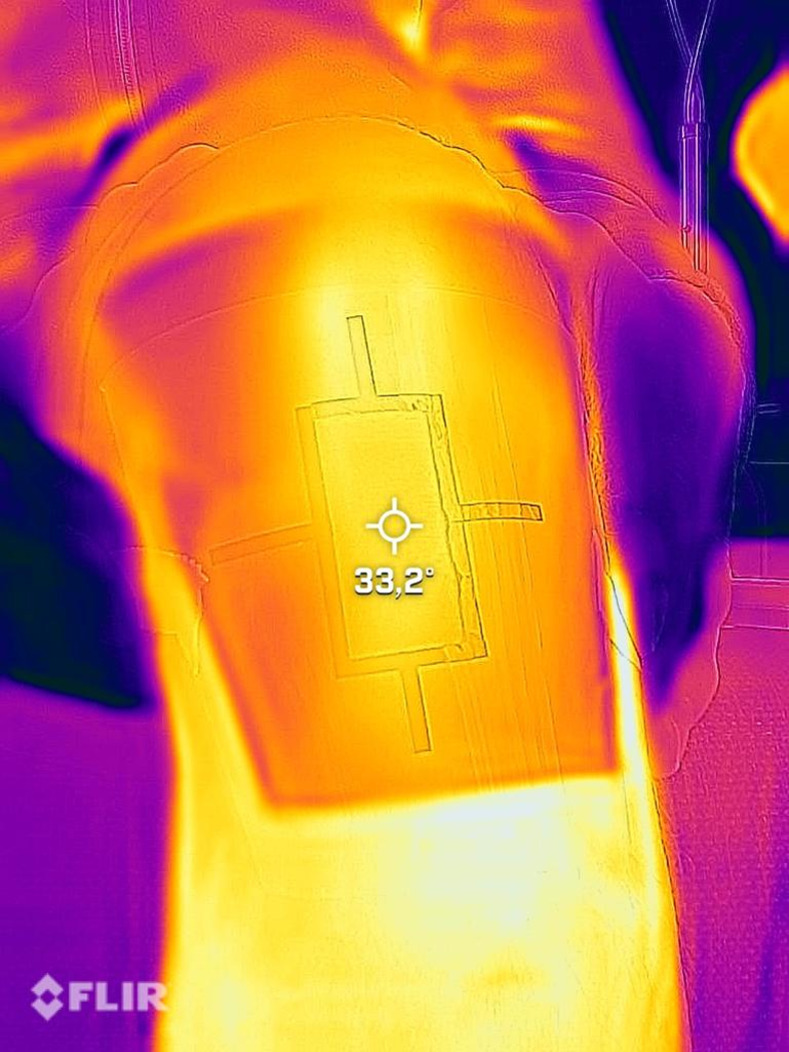
Thermography of the patellar tendon.

Temperature measurement using thermography covers temperature ranges from -20° to 400° Celsius (Flir One, Teledyne FLIR LLC, Wilsonville, US). Measurement accuracy is +/- 3° or +/- 5%. This is valid 60 seconds after switching on the instrument in an ambient temperature of 15° to 35° Celsius and the target temperature is in the range of 5° and 120° Celsius. To ensure measurement at a reproducible measuring point, the template (Fig 2A) was provided with a marker that ensured exact positioning of the image section for temperature determination (Fig 4). The mean of three measurements was calculated for each measurement time point.

### Statistics

The standard deviation (SD), mean, and 95% confidence interval (95% CI) were determined for all parameters. There were no outliers in the data. The outcome variables were normally distributed as determined by the Shapiro-Wilk test (p > 0.05). The homogeneity of the error variances between the groups was fulfilled for all these variables according to Levene’s test (p > 0.05). Differences between measurements were tested for significance using a repeated measures ANOVA. For significant main effects, pairwise comparisons were performed with Bonferroni correction. The effects of the regression to the mean were calculated and the treatment effects were adjusted respectively according to Barnet et al. [24]. The significance level was set at p = 0.05.

Libreoffice Calc version 6.4.7.2 (Mozilla Public License v2.0) was used for the descriptive statistics. The inferential statistics were carried out with the software R, version 3.4.1 (R Foundation for Statistical Computing, Vienna, Austria).

## Results

The anthropometric data and baseline characteristics are shown in Table 1. Of 28 participants screened between 18/02/2022 and 30/03/2022, 21 met eligibility criteria and were enrolled in the study. None of the participants reported any adverse side effects of HILT, discontinued treatment, or were lost to follow-up (Fig 1).

**Table 1:** Baseline characteristics.

Baseline characteristics	Participants (n = 21) mean ± SD
Sex (men/woman)	15/6
Age (years)	22.1 ± 4.2
Height(m)	1.75 ± 0 .1
Weight (kg)	76.0 ± 11.5
BMI (kg/m^2^)	24.9 ± 3.5
Sport activity (hours per week)	6.0 ± 4.2

SD standard deviation. n number.

**Fig 5:**
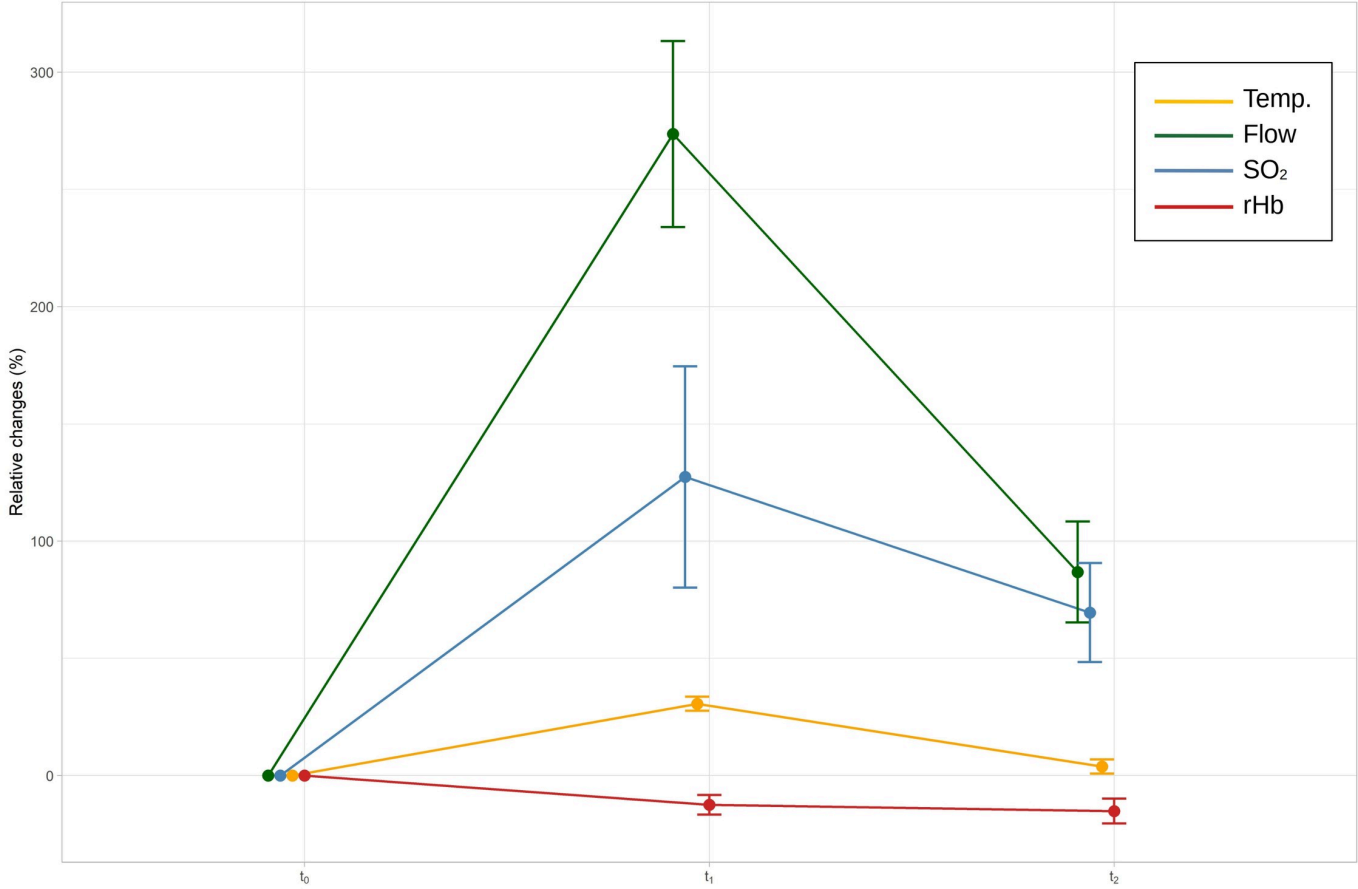
Changes between baseline (t_0_), after treatment (t_1_), and 10 minutes later (t_2_). AU, arbitrary units; Temp, temperature; SO_2_, oxygen saturation; rHb, relative hemoglobin concentration. Error bars show the 95% confidence interval.

A repeated-measures ANOVA revealed that blood flow, F(1.56, 31.23) = 116.15, p < 0.001, partial η² = 0.85, and SO_2_, F(2, 40) = 25.01, p < 0.001, partial η² = 0.56, as well as rHb, F(2, 40) = 7.24, p = 0.002, partial η² = 0.27, showed statistically significant differences between measurements. There was also a significant difference for skin temperature, F(2, 40) = 342.46, p < 0.001, partial η² = 0.96. Table 2 provides the descriptive statistics for the results, and Table 3 shows the Bonferroni-adjusted post-hoc analysis with further correction for the effects of regression to the mean. Fig 5 provides an overview of the relative changes.

**Table 2:** Descriptive statistics.

Outcome	*n*	Mean ± SD (95% CI)	Mean ± SD (95% CI)	Mean ± SD (95% CI)
Flow (AU)	21	38 ± 19 (30.7–47.2)	123 ± 35 (111.3–139.3)	65 ± 24 (53.4–76.1)
SO_2_ (%)	21	37 ± 16 (28.4–44.0)	57 ± 20 (46.8–66.0)	51 ± 17 (41.0–58.4)
rHb (AU)	21	45 ± 10 (39.5–48.2)	38 ± 7 (34.2–40.2)	36 ± 8 (32.5–39.4)
Temperature (°C)	21	28.2 ± 1.1 (27.7–28.5)	37.7 ± 1.7 (36.9–38.5)	30.2 ± 1.3 (29.6–30.8)

SD, standard deviation; n, number; AU, arbitrary units; SO2, oxygen saturation; rHb, relative hemoglobin concentration.

95% CI, 95% confidence interval.

**Table 3:** Pairwise comparisons and adjustment for regression to the mean.

Outcome	Time	*n*	ΔMean	ΔMean_adj_	SE	df	t	P-value^*^
Flow (AU)	t_0_ –t_1_	21	-86.40	-86,37	7.08	20.0	-12.21	<0.001
	t_0_ –t_2_	21	-25.80	-25.76	4.37	20.0	-5.90	<0.001
	t_1_ –t_2_	21	60.60	60.56	5.70	20.0	10.64	<0.001
SO_2_ (%)	t_0_ –t_1_	21	-20.14	-19.98	3.23	20.0	-6.24	<0.001
	t_0_ –t_2_	21	-13.48	-13.32	2.58	20.0	-5.23	<0.001
	t_1_ –t_2_	21	6.67	6.51	2.86	20.0	2.33	0.092
rHb (AU)	t_0_ –t_1_	21	6.67	6.50	2.30	20.0	2.903	0.026
	t_0_ –t_2_	21	7.90	7.73	2.42	20.0	3.270	0.011
	t_1_ –t_2_	21	1.24	1,07	1.97	20.0	0.630	0.999
Temperature (°C)	t_0_ –t_1_	21	-9.60	-8.03	0.402	20.0	-23.88	<0.001
	t_0_ –t_2_	21	-2.10	-1.07	0.318	20.0	-6.60	<0.001
	t_1_ –t_2_	21	7.50	6.47	0.452	20.0	16.60	<0.001

SD, standard deviation; n, number; AU, arbitrary units; SO2, oxygen saturation; rHb, relative hemoglobin concentration; ΔMean, mean difference; ΔMean_adj_, adjusted mean difference for regression to the mean.

^*^ P-values were adjusted with Bonferroni correction.

## Discussion

In this study, the influence of HILT on patellar tendon microcirculation and skin temperature was investigated. The results here showed significant changes in all measured parameters.

Blood flow increased both immediately after intervention and at follow-up (86.38 AU and 25.76 AU, respectively). These values not only showed statistical significance, they also exceeded the minimum detectable changes of 9.2 AU determined by Wezenbeek et al. [8], although the authors determined these values on the Achilles tendon and the transferability to the patellar tendon is debatable. Furthermore, an increase in SO_2_ was also observable, which was 20.14% after the intervention and 13.48% at follow-up. This improvement of the capillary blood supply, which we hypothesized, could follow a vasodilatation by thermal influences [25]. However, this should also have caused an increase in rHb. In a study preceding this one, which investigated the influences of purely local thermal changes on the patellar tendon on the microcirculation, an increase in rHb was observed [23]. Nevertheless, the results of this study show a different picture, which is why HILT-specific mechanisms of influence can also be discussed here.

Skin thermal changes, which were recorded by infrared thermography followed the blood flow and were as expected. Immediately after HILT application, the temperature increased by 9.45° Celsius and at follow-up by 1.94° Celsius compared to baseline measurement.

In laboratory studies, there was some evidence of changes in erythrocytes and platelets due to laser therapy that significantly affected flow characteristics. Thereby, the blood viscosity decreased and certain stress adaptation of erythrocytes occurred [2,18,19]. The phenomenon of an apparent decrease in relative hemoglobin observed in the study would also be consistent with this. The rHb value was decreased by 6.67 AU after the intervention and by 7.9 AU at follow-up. To the authors’ knowledge, this response has not been observed in other studies examining only heat-dependent effects and may indicate improved microcirculatory flow properties that increased blood flow velocity in the venous-capillary vasculature. This would explain that the O2C device detected a faster clearance of erythrocytes, leading to a decrease in rHb [26]. However, it should be critically noted in these considerations that although statistically significant results are available here, the observed value changes are relatively small, and it seems questionable whether the minimum detectable changes were exceeded.

Recent studies have also identified red cell changes in long-COVID-19 patients that could affect microcirculation and lead to typical long-COVID-19 symptoms such as fatigue and muscle weakness [27,28]. Therefore, in addition to the scattered work on laser therapy as a method to treat long-COVID-19, work focusing on HILT-specific enhancement of erythrocyte flow properties is of great interest here [3,20].

The study had the strengths mentioned above, but some limitations should be taken into account. For example, it was conducted in an experimental design that was unblinded, not randomized and not placebo-controlled. It was tried to address this in the design. Multiple measurements at each measurement time point and the use of a 10 second time series to derive blood flow data may have reduced the risk of regression to the mean. However, further high-quality studies are needed to validate these experimental results. In particular, the presumed laser-specific results need to be validated with a second intervention group receiving only heat therapy, in addition to a control group. Moreover, the study group was relatively young, physically active, and of normal weight. Therefore, transferability of the study results to other populations is limited. The results should be understood under the premise of a basic scientific study.

Significant changes in blood flow could be detected after HILT application, which also exceeded the minimal detectable changes. Here it may have an effect beyond mere vasodilation, improving the flow properties of erythrocytes and platelets. Therefore, HILT could be a valuable additional treatment option for clinicians in tendon disorders with impaired microcirculation. Furthermore, it could also be useful in the prevention of sports-related tendon overload associated with a decrease in blood flow. In any case, further studies on the interrelationships of laser-specific therapeutic effects are needed to provide valid clinical recommendations for use.

## Conclusion

In this study, the effects of HILT on the microcirculation of the patellar tendon and skin temperature were investigated. It was found a significant improvement in blood flow and oxygen saturation after HILT, which were still detectable 10 minutes later. On the other hand, the relative amount of hemoglobin decreased significantly, which could indicate an acceleration of blood flow due to improved flow properties of erythrocytes and platelets. Skin temperature followed changes in blood flow and oxygen saturation. HILT may be a useful therapeutic option for tendon pathologies with impaired microcirculation, but further high-quality studies are needed to validate these experimental results.

## Supporting information

S1 Checklist STROBE statement—checklist of items that should be included in reports of observational studies. (PDF)

S1 Data (XLSX)

S1 File (PDF)

S2 File (PDF)
