# Supplementary material for: Influence of high-energy laser therapy to the patellar tendon on its ligamentous microcirculation: An experimental intervention study
Source: PLoS One. 2023 Mar 27;18(3):e0275883. doi: 10.1371/journal.pone.0275883 (PMC10042339; doi:10.1371/journal.pone.0275883)
Supplement: S1 File — (PDF) [file pone.0275883.s003.pdf]

Version 2
Diploma University 16.12.2021

Ethics Consulting
Am Hegeberg 2
D-37242 Bad Sooden-Allendorf

Application to the Ethics Commission of the Diploma University for the Ethical Assessment of a Medical Research Project in Humans

Submitted by:

Prof. Dr. Christoph Egner
Thomas Gutmann M.Sc.
Dr Robert Schleip
Andreas Brandl M.Sc.

Contact: christoph.egner@diploma.de

Study projects:

Influence of high-energy laser light application to the patellar tendon on the ligamentary microcirculation. A quasi-experiment.

NOTE: The following application is based on the checklist of ethics advice of the Diploma University (https://www.fh-diploma.de/mod/resource/view.php?id=48915)

Content

1 Study protocol ... 33

1.1 Title of the study ... 33

1.2 Background and objectives of the study ... 33

1.3 Characteristics of study participants ... 3

1.4 Recruitment/Field Access ... 43

1.5 Study design ... 43

1.6 Course of study ... 43

1.7 Measures ... 44

1.7.1 Microcirculation ... 44

1.7.2 Infrared surface temperature ... 55

1.7.3 Thermography ... 55

1.8 Questions ... 55

1.9 Disturbances ... 66

1.10 Biometric evaluation ... 66

1.11 Organisation of studies ... 66

1.12 Data protection ... 66

1.13 Patient safety ... 66

1.14 Quality management ... 77

2. Information for study participants ... 88

3. Declaration of consent ... 1010

4. Checklist for study participation ... 1011

5 Literature ... 1212

1 Study protocol

1.1 Title of the study

Influence of high-energy laser light application to the patellar tendon on its ligamentary microcirculation. A quasi-experiment.

1.2 Background and objectives of the study

Electrotherapeutic applications in the context of physical therapy are mandatory to provide proof of efficacy against the background of finite financial resources of the health system. In the field of empirical research, there is a deficit with regard to fascial structures, their variability and adaptability (Willard et al., 2012). Non-invasive therapy devices are becoming increasingly important in the context of musculoskeletal rehabilitation. High-intensity laser therapy (HILT) has so far been little investigated in previous studies as an intervention method for knee osteoarthritis (Wyszyńska et Bal-Bocheńska, 2018). Basic studies on the mechanisms of action of HILT on ligamentary tissue are scarcely available.

Some authors report an improvement in microvascularization as one of the histological effects after laser treatments (Tumilty et al., 2010; Kulchitskaya et al., 2016).. In addition,an acceleration of blood flow through improved microcirculation after the application of laser therapy has been confirmed in numerous n works (Mussttaf et al., 2019; Hamblin et al., 2017; Tkocz et al., 2021; Kulchitskaya et al., 2016).. In addition to the thermal effects of HILT therapy, photobiomodulatory effects are also described (Hamblin et al., 2017).

Kulchitskaya et al. (2016) investigated the effect of HILT on microcirculation in patients with knee arthritis. They applied pulsed HILT with a wavelength of 1064 nm (25 Hz, 10 J/cm 2) to a group of 30 study participants for 4 minutes each in the area of the joint gap and in the popliteal region. Using laser Doppler flowmetry, they were able to detect a significant improvement in endothelial function ($p<0.001$), a normalization of the muscle tone of the arterioles ($p<0.05$), a reduction in the neurogenic tone of the arterioles ($p<0.01$) and an increase in capillary blood filling in the venous part of the microcirculatory bloodstream.

Since the aforementioned work gives a strong indication of a positive influence on the microcirculation according to HILT, the study should in particular investigate the questions of whether similar changes can also be detected in the tissue of the patellar tendon and , if necessary, confirmed whether these correlate with a heat effect or whether an alternative mechanism could be underlying.

The main objective of the study is to investigate microcirculatory changes in tendon tissue caused by HILT based on a bachelor's thesis (Spieß, 2021) carried out in the winter semester 2020/21 at the Diploma Hochschule.

1.3 Characteristics of study participants

A group size of 20 healthy volunteers in the age range of 18 to 50 years is sought. The inclusion criteria are: a generally healthy constitution, no mental illness, no operations in the last three months, no prosthetics or knee replasties, acute inflammation, large scar area, knowing adhesions, skin diseases or hematomas in the area of the knee joint. The persons to be treated must have an intact thermal sensation and be able to perceive and communicate pain. As a result, people cannot participate when taking painkillers or mind-altering substances. These criteria are queried by the volunteers in a checklist (see Annex 4) with individually answered questions in the run-up to the study participation.

1.4 Recruitment/Field Access

The participants of this study are bachelor students or trainees in physiotherapy, as well as volunteers from the environment of the Diploma University at the Bad Sooden-Allendorf location. The voluntariness is again communicated by the investigators to each participating person before the collection of the first data, as well as before each measurement: *"I inform you again that the study participation is absolutely voluntary and you have no disadvantages to expect if you end the participation now."*

1.5 Study design

The planned work is a quasi-experiment within the framework of basic research.

1.6 Course of study

First, the anthropometric data, age, gender, height and weightof the test lead are collected.

Before the measurements, the subjects are given information regarding the implementation of the intervention and measurements. You sit on an upholstered chair with a backrest on the side next to a treatment table. The sitting position should be comfortable and imply a hip flexion of 120° and a knee flexion of 110°.

First, the input measurement (baseline) is carried out. HilT (OptonPro© 25 Watt – 810 nm, 980 nm, 1064 nm, Zimmer Medizinsysteme GmbH, Neu-Ulm) is then applied to the randomly selected (right or left, determined by lot) patellar tendon for 2 minutes (Fig. 1). A post-interventional measurement is carried out immediately after the procedure, as well as 10 minutes later.

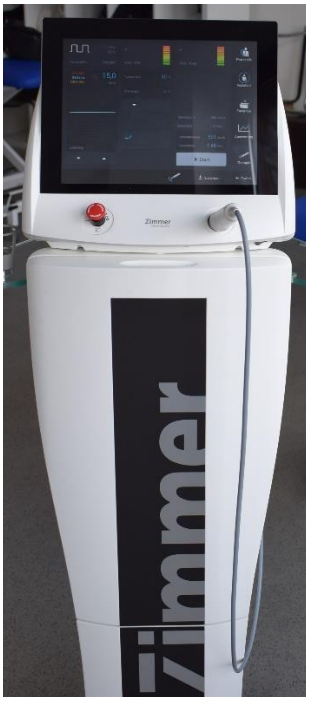

Figure 1. OptonPro High-Energy Vice Therapy Device

1.7 Measures

1.7.1 Microcirculation

The ligamentary microcirculation of the patellar tendon is measured using laser Doppler flowmeters and tissue spectrometers (O2C, LEA Medizintechnik GmbH, Heuchelheim) (Fig. 2). This makes it possible to simultaneously determine blood flow velocity, blood flow, sowsaturation and relative

hemoglobin amount of the measuring tissue at different measuring depths. It determines these parameters at the venous end of the capillary and thus provides information about local metabolism, i.e. measures the local microcirculation. It is a possibility of non-invasive diagnosis of microperfusion.

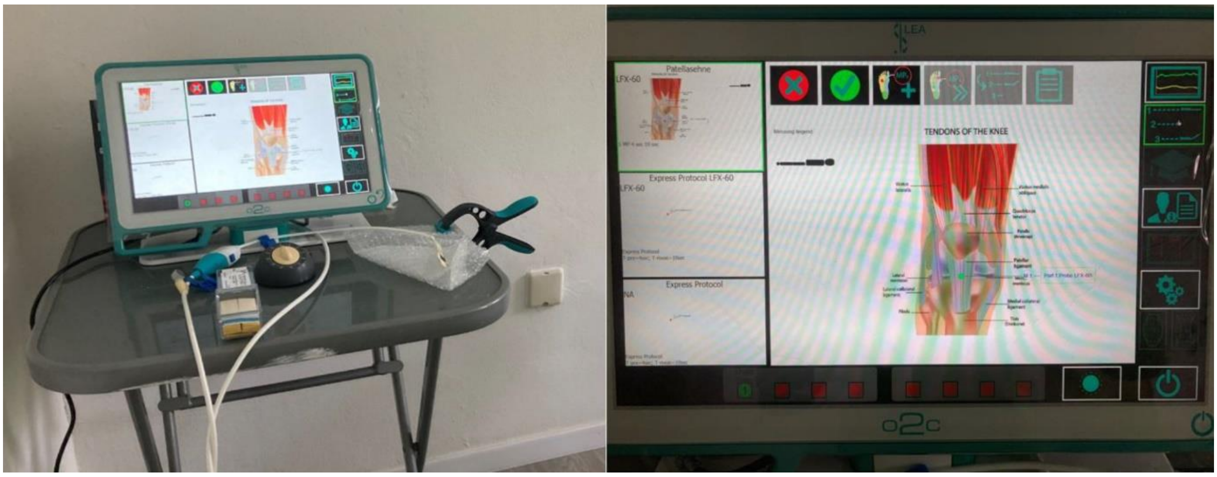

Figure 2. Hardware for microcirculation measurement using laser Doppler flowmeters and tissue spectrometers

1.7.2 Infrared surface temperature

This is a contactless infrared thermometer (Beurer GmbH, Ulm). It is battery powered and has a digital display. The thermometer is able to measure temperatures of surfaces, objects and liquids. The measurement accuracy for objects/surfaces is +/-3° Celsius (+/- 0.5°F). The measuring range is -22° Celsius to 80° Celsius.

1.7.3 Thermography

Temperature measurement using thermography captures temperature ranges from -20° Celsius to 400° Celsius (Flir One, Teledyne FLIR LLC, Wilsonville, US). To use the camera it requires the manufacturer app which is available free of charge. By means of this it is possible to organize image recordings in galleries and to store these files externally. Each image file shows the recorded temperature range. The measurement accuracy +/- 3° Celsius or +/- 5%. This applies 60 seconds after switching on the device in an ambient temperature of 15° Celsius to 35° Celsius and the target temperature is in the range of 5° Celsius and 120° Celsius. Several markers can also be set in the image. These markers show an exact number of degrees. In the intended experiment, two markers, once in the middle of the patellar tendon and an area showing an average temperature, are recorded.

1.8 Questions

Previous studies on HILT and its effect on microcirculation or micro-metabolism are absent or of low quality (Kulchitskaya et al., 2016; Angelova et Ilieva, 2016; Wyszyńska et Bal-Bocheńska, 2018).. The aim of the study is therefore to contribute to basic research in this field. To this end, the following research questions are established:

- Does HILT treatment of the patellar tendon achieve significant (and/or clinically relevant) temperaturechanges in it?
- Can HILT treatment of the patellar tendon cause significant (and/or clinically relevant) changes in microcirculation?

1.9 Disturbances

Room temperature and lighting conditions could be critical confounders for the measurements. Particular attention must be paid to this during the conduct of the study and precisely reproducible spatial conditions must be ensured.
Another critical variable could be the age of the subject, as the microcirculation is influenced by aging processes. This influence on the measurement should be avoided by appropriate statistical consideration.

1.10 Biometric evaluation

It is assumed that there is no normal distribution of the variables based on the sample size and study design. For all parameters, the median, first and third quartiles are given, as well as significance testing using the Wilcoxon-Mann-Whitney test as a non-parametric alternative to the Students t-test.

The program Microsoft Excel 2019 (Microsoft Corporation) is used for data management and the calculation of descriptive statistics . The statistics program R Version 3.4.1 (Foundation for Statistical Computing) is used to calculate the inference statistics. The significance level is set to p= 0.05.

1.11 Organisation of studies

It is a monocentric study that is to be carried out in the premises of the Diploma Hochschule, Bad Sooden-Allendorf. The study is led by Prof. Dr. Christoph Egner, further contributors are Dr. Robert Schleip, Thomas Gutmann and Andreas Brandl. The study does not receive any external financial support or is carried out with its own resources.

The start of studies is planned for 10.01.2022, but at the earliest after the ethics vote has been issued by the Ethics Advisory Service of the Diploma University and registration in the German Register of Clinical Studies (DRKS). The planned duration of the data collection is 12 weeks.

1.12 Data protection

The applicable legal data protection regulations (General Data Protection Regulation, GDPR) are complied with. The data collection takes place anonymously. This also includes the storage of image and video material. After evaluation using the Advanced Encryption Standard block cipher method, the data is stored on a 25 gigabyte blue-ray disc with a 128-bit key, password-protected (at least 20 characters, upper and lower case letters, special characters and digits), of which a backup copy is created. A disc remains in the Diploma Hochschule, the backup is stored in a fireproof safe in Andreas Brandl's practice. Personalized data will not be collected and will not be passed on to third parties.

1.13 Patient safety

Before the start of the study, all participants are informed about the contents, goals and measurement methods in a conversation. They will also be informed in writing and must express their willingness to participate by their signature.

The laser used is a class 4 laser. This laser radiation is very dangerous for the eye and dangerous for the skin. Diffusely scattered radiation can also be dangerous. The Occupational Health and Safety Ordinance on Artificial Optical Radiation (OStrV) applies. Accordingly, a laser safety officer is appointed by the operator, who instructs the users in the use of the device and safety. These have the legal requirements for the application of medical laser treatment on humans. The operating range of the laser and all doors to the operating area are provided with laser warning signs. All persons in the treatment room must wear laser safety goggles with an optical density OD>3 (protection level at least LB 3) at 810/980/1064 nm for operating mode D (continuous wave) and a light transmission of at least 20% in the visible range. The glasses must be both heat and UV resistant and meet the

requirements of EN 207. The safety goggles must be designed for the power of 25 W. The user and subject must not wear reflective and scattering objects in the treatment field, such as rings and piercings.

All participants are constantly supervised during the examination and monitored by an experienced therapist instructed in the use and safety of the OptonPro (>10 years in manual therapy, doctorate or M.Sc degree). In addition, an emergency number will be issued if adverse events occur outside the university rooms after participating in the study.

The aim is to register with the German Register of Clinical Trials after the positive ethics vote of the Diploma University. The study is consistent with the Declaration of Helsinki in its current version (World Medical Association, 2021).

1.14 Quality management

The study is carried out in accordance with the planned measurement procedures in accordance with standardized procedures. The test management istrained according to the requirements. Quality-related parameters are checked after each measurement of a participant. This is logged and documented accordingly. Error prevention measures with regard to tape handling and potential measurement errors have been developed and can be correctively applied in the ongoing study.

2. Information for study participants

Test person information on the study

"Influence of high-energy laser light application to the patellar tendon on its ligamentary microcirculation. A quasi-experiment. "

Dear Subject,

Thank you for agreeing to participate in this study. Below you will find some background information on the necessity and purpose of this work.

Purpose of the study

Non-invasive therapy devices are becoming increasingly important in the treatment and rehabilitation of diseases of the musculoskeletal system. High-energy laser therapy is a relatively new method. So far, however, little is known about the mechanisms of action behind this form of therapy. It is of particular scientific interest to investigate these relationships in more detail. In this study, measurements are to be carried out on healthy people and data collected in order to gain insights into how laser therapy on the tendon below the kneecap affects blood circulation.

Courseof studies

An appointment (about 45 minutes) will be arranged with you. After an informational interview, thetemperature and blood flow to your tendon below the kneecap are measured. This is done on the skin and is painless. Subsequently, the tendon is treated by means of a high-energy laser light. During this treatment, you must wear safety glasses that shield your eyes from laser beams. You must not wear reflective or scattering objects such as rings or piercings in the treatment area. This treatment is also contactless and generally painless. Immediately afterwards and 10 minutes later, the temperature and blood flow of the tendon is measured again.

The Covid-19 pandemic requires special protection for the subjects in this study, so the current hygiene guidelines are adhered to throughout the study.

Possible risks

Laser therapy has no side effects when used correctly. During the laser light treatment, however, in some cases there may be an unpleasant sensation of temperature and rarely also in the course of it a pain caused by punctual heat. Here there is always the possibility to stop the treatment, the laser light is then switched off immediately, so that another pain effect is abruptly prevented.

Personal benefits of the study

By participating in the study, you are making a valuable contribution to research in the human sciences. With your help , basic data on the effect of laser light therapy can be collected in order to be able to treat connective tissue and muscle diseases more effectively in the future. There is no immediate advantage for you.

Criteria

They must be between 18 and 50 years old and have a generally healthy constitution. Unfortunately, you cannot participate if you suffer from pre-existing mental illnesses, acute inflammation or extensive scars on the knee joint. You should have no surgery in the last three months, no knee joint prosthesis, knowing adhesions or hematomas in this area.

To clarify these conditions of participation, a checklist for your personal use is available.

Cost

Of course, you will not incur any costs for participating in the study.

Voluntary participation and right of withdrawal

Participation in the study is voluntary. You may terminate your participation in this study at any time and without giving reasons, without incurring any disadvantages for you. Even if you stop the study prematurely, you will not have any disadvantages.

Confirmation of confidentiality and data protection

The personal data collected in the context of the study after a declaration of consent are subject to confidentiality and the provisions of the data protection law. They are recorded in paper form and kept in the care of the Diploma University. The use of the data takes place in encrypted form, i.e. the determined data is not stored under your name, but under an anonymous numerical code. A transfer of the collected data within the scope of the research purpose takes place only in encrypted form. The same applies to the publication of the study results.

The recording or storage takes place for a period of 10 years.

3. Declaration of consent

Declaration of consent of the patient to the study

"Influence of high-energy laser light application to the patellar tendon on its ligamentary microcirculation. A quasi-experiment."

Name:____________
First name:_______
Date of birth:____________

I, _____ I have read and understood the Enlightenment text. I have had the opportunity to ask questions and have understood and accept the answers. The investigator informed me about the risks and potential benefits associated with participating in the study.

I had enough time to decide to participate in this study and know that participating in this study is voluntary. I know that I can revoke this consent at any time and without giving reasons, without this decision having a detrimental effect on the later treatment by my therapist.

I am aware that this declaration of consent is transferred to the care of the Diploma University and is kept by it in accordance with the provisions of the Data Protection Act.

Checklist for study participation

Scientific experiments and studies must be carried out under comprehensible conditions with as uniform a group of study participants as possible. If you answer **"yes"** to one of the following questions , you will unfortunately not be able to participate in the study, which of course is not a personal shortcoming, but is due to the requirements for quasi laboratory conditions.

☐ **yes** ☐ **no** You have not yet reached the age of 18 or have already reached the age of 49.

☐ **yes** ☐ **no** You've had surgery in the last three months.

☐ **yes** ☐ **no** You know about adhesions, hematomas or skin diseases in the area of the knee joint (knee joint and 10 cm each of the adjacent upper or lower leg).

☐ **yes** ☐ **no** You suffer from acute or chronic inflammation of the knee joint.

☐ **yes** ☐ **no** You have been in psychotherapeutic and/or psychiatric treatment for the last two years
.

☐ **yes** ☐ **no** You have larger scars (2 cm long and larger) in the area of the knee joint (knee joint and 10 cm each of the adjacent upper or lower leg).

☐ **yes** ☐ **no** You have had a knee joint prosthesis inserted.

☐ **yes** ☐ **no** **You have kan intact thermal Eand can** not **perceive and communicate** pain **or reduced**.

☐ **yes** ☐ **no** **You take painkillers or mind-altering substances.**

With my consent to participate, I declare that I have answered the checklist for study participation to the best of my knowledge and that I agree to the anonymous recording of examination and disease data within the framework of this study.
I have received a copy of the study information and this declaration of consent. I hereby declare my voluntary participation in this study.

____
____
Place and date

Signature of the patient /
of the patient

____
Place and date

____
Signature of the enlightening
Test management

5 Literature

Angelova, A., Ilieva, E. M. (2016). *Effectiveness of High Intensity Laser Therapy for Reduction of Pain in Knee Osteoarthritis*. Pain Research and Management 2016, 1–11.

Hamblin, M., 1 Wellman Center for Photomedicine, Massachusetts General Hospital, BAR414, 40 Blossom Street, Boston, MA 02114, USA, 2 Department of Dermatology, Harvard Medical School, Boston, MA 02115, USA, 3 Harvard-MIT Division of Health Sciences and Technology, Cambridge, MA 02139, USA (2017). *Mechanisms and Applications of the Anti-Inflammatory Effects of Photobiomodulation*. AIMS Biophysics 4 (3), 337–361.

Kulchitskaya, D. B., Konchugova, T. V., Fedorova, N. E. (2016). *Comparative evaluation of the effects of high- intensity and low-intensity laser radiation on microcirculation among patients with knee arthritis*. Journal of Physics: Conference Series 755, 011001.

Mussttaf, R. A., Jenkins, D. F. L., Jha, A. N. (2019). *Assessing the Impact of Low Level Laser Therapy (LLLT) on Biological Systems: A Review*. International Journal of Radiation Biology 95 (2), 120–143.

Spieß, C. (2021). *Investigation of the influence of induced temperature changes on the blood circulation of the patellar tendon*. Diploma University.

Tkocz, P., Matusz, T., Kosowski, Ł., Walewicz, K., Argier, Ł., Kuszewski, M., Hagner-Derengowska, M., Ptaszkowski, K., Dymarek, R., Taradaj, J. (2021). *A Randomised-Controlled Clinical Study Examining the Effect of High-Intensity Laser Therapy (HILT) on the Management of Painful Calcaneal Spur with Plantar Fasciitis*. Journal of Clinical Medicine 10 (21), 4891.

Tumilty, S., Munn, J., McDonough, S., Hurley, D. A., Basford, J. R., Baxter, G. D. (2010). *Low Level Laser Treatment of Tendinopathy: A Systematic Review with Meta-Analysis*. Photomedicine and Laser Surgery 28 (1), 3–16.

Willard, F. H., Vleeming, A., Schuenke, M. D., Danneels, L., Schleip, R. (2012). *The Thoracolumbar Fascia: Anatomy, Function and Clinical Considerations*. Journal of Anatomy 221 (6), 507–536.

Wyszyńska, J., Bal-Bocheńska, M. (2018). *Efficacy of High-Intensity Laser Therapy in Treating Knee Osteoarthritis: A First Systematic Review*. Photomedicine and Laser Surgery 36 (7), 343–353.
