# Supplementary material for: Influence of high-energy laser therapy to the patellar tendon on its ligamentous microcirculation: An experimental intervention study
Source: PLoS One. 2023 Mar 27;18(3):e0275883. doi: 10.1371/journal.pone.0275883 (PMC10042339; doi:10.1371/journal.pone.0275883)
Supplement: S2 File — (PDF) [file pone.0275883.s004.pdf]

An die
Diploma Hochschule
Ethikberatung
Am Hegeberg 2
37242 Bad Sooden-Allendorf

Version 2
16.12.2021

Antrag an die Ethik-Kommission der Diploma Hochschule zur ethischen Begutachtung eines medizinischen Forschungsvorhabens am Menschen

Eingereicht von:

Prof. Dr. Christoph Egner
Thomas Gutmann M.Sc.
Dr. Robert Schleip
Andreas Brandl M.Sc.

Kontakt: christoph.egner@diploma.de

Studienvorhaben:

Einfluss von hochenergetischer Laserlichtapplikation an der Patellarsehne auf deren ligamentäre Mikrozirkulation. Ein Quasi-Experiment.

HINWEIS: Nachfolgender Antrag orientiert sich inhaltlich an der Checkliste der Ethik-Beratung der Diploma Hochschule (https://www.fh-diploma.de/mod/resource/view.php?id=48915)

Inhalt

1 Studienprotokoll 3
1.1 Titel der Studie 3
1.2 Hintergrund und Zielsetzung der Studie 3
1.3 Eigenschaften der Studienteilnehmenden 3
1.4 Rekrutierung/Feldzugang 4
1.5 Studiendesign 4
1.6 Studienablauf 4
1.7 Messgrößen 4
1.7.1 Mikrozirkulation 4
1.7.2 Infrarot-Oberflächentemperatur 5
1.7.3 Thermographie 5
1.8 Fragestellungen 5
1.9 Störgrößen 6
1.10 Biometrische Auswertung 6
1.11 Studienorganisation 6
1.12 Datenschutz 6
1.13 Patientensicherheit 6
1.14 Qualitätsmanagement 7
2. Informationen für Studienteilnehmende 8
3. Einwilligungserklärung 10
4. Checkliste zur Studienteilnahme 10
5 Literatur 12

1 Studienprotokoll

1.1 Titel der Studie

Einfluss von hochenergetischer Laserlichtapplikation an der Patellarsehne auf deren ligamentäre Mikrozirkulation. Ein Quasi-Experiment.

1.2 Hintergrund und Zielsetzung der Studie

Elektrotherapeutische Anwendungen im Rahmen der physikalischen Therapie sind vor dem Hintergrund endlicher finanzieller Ressourcen des Gesundheitssystems in der Pflicht Wirksamkeitsnachweise zu erbringen. Im Bereich der empirischen Forschung existiert ein Defizit bezüglich faszialer Strukturen, deren Variabilität und Adaptionsfähigkeit (Willard et al., 2012). Nicht-invasive Therapiegeräte erlangen im Rahmen der muskuloskelettalen Rehabilitation immer wichtigere Bedeutung. Die hochenergetische Lasertherapie (high-intensity laser therapy, HILT) wurde bisher nur wenig in vorherigen Studien als Interventionsmethode bei Knieosteoarthritis untersucht (Wyszyńska et Bal-Bocheńska, 2018). Grundlagenstudien über die Wirkmechanismen von HILT auf ligamentäres Gewebe sind kaum vorhanden.

Einige Autoren berichten von einer Verbesserung der Mikrovaskularisation als einen der histologischen Effekte nach Laserbehandlungen (Tumilty et al., 2010; Kulchitskaya et al., 2016). Darüber hinaus wurde in zahlreichen Arbeiten eine Beschleunigung des Blutflusses durch eine verbesserte Mikrozirkulation nach der Anwendung von Lasertherapie bestätigt (Mussttaf et al., 2019; Hamblin et al., 2017; Tkocz et al., 2021; Kulchitskaya et al., 2016). Neben den thermalen Effekten der HILT-Therapie werden auch photobiomodulatorische Effekte beschrieben (Hamblin et al., 2017).

Kulchitskaya et al. (2016) untersuchten die Wirkung von HILT auf die Mikrozirkulation bei Patienten mit Kniearthritis. Sie applizierten einer Gruppe von 30 Studienteilnehmenden gepulste HILT mit einer Wellenlänge von 1064 nm (25 Hz, 10 J/cm^2) für jeweils 4 Minuten im Bereich des Gelenkspaltes und in der Poplitealregion. Dabei konnten sie mittels Laser-Doppler-Flowmetrie eine signifikante Verbesserung der Endothelfunktion ($p<0.001$), eine Normalisierung des Muskeltonus der Arteriolen ($p<0.05$), eine Verringerung des neurogenen Tonus der Arteriolen ($p<0.01$) und eine Steigerung der Kapillarblutfüllung im venösen Teil des mikrozirkulatorischen Blutstromes feststellen.

Nachdem die vorgenannten Arbeiten einen starken Hinweis auf eine positive Beeinflussung der Mikrozirkulation nach HILT geben, soll die Studie insbesondere den Fragen nachgehen, ob ähnliche Veränderungen auch im Gewebe der Patellarsehne nachzuweisen und ggf. zu bestätigen sind, ob diese ggf. mit einer Wärmewirkung korrelieren oder ob ein alternativer Mechanismus zugrunde liegen könnte.

Hauptziel der Studie ist es, aufbauend auf einer im Wintersemester 2020/21 in der Diploma Hochschule durchgeführten Bachelorarbeit (Spieß, 2021), mikrozirkulatorische Veränderungen des Sehnengewebes durch HILT zu untersuchen.

1.3 Eigenschaften der Studienteilnehmenden

Es wird eine Gruppengröße von 20 gesunden Freiwilligen im Altersbereich von 18 bis 50 Jahren angestrebt. Als Einschlusskriterien gelten: eine allgemein gesunde Konstitution, keine psychischen Vorerkrankungen, keine Operationen in den letzten drei Monaten, keine Prothetik oder Kniebinnenplastiken, akute Entzündungen, großes Narbengebiet, wissentliche Verwachsungen, Hauterkrankungen oder Hämatome im Bereich des Kniegelenkes. Die zu behandelnden Personen müssen über ein intaktes thermisches Empfinden verfügen und Schmerzen wahrnehmen und kommunizieren können. Demzufolge können Personen bei Einnahme von Schmerzmitteln oder bewusstseinsverändernden

Substanzen nicht teilnehmen. Diese Kriterien werden in einer Checkliste (siehe Anhang 4) mit individuell zu beantwortenden Fragen im Vorfeld der Studienteilnahme von den Freiwilligen abgefragt.

1.4 Rekrutierung/Feldzugang

Die Teilnehmenden dieser Studie sind Bachelorstudenten oder Auszubildende der Physiotherapie, sowie Freiwillige aus dem Umfeld der Diploma Hochschule am Standort Bad Sooden-Allendorf. Die Freiwilligkeit wird nochmals von den Untersuchenden gegenüber jeder teilnehmenden Person vor Erhebung der ersten Daten, sowie vor jeder Messung kommuniziert: *„Ich informiere Sie nochmals, dass die Studienteilnahme absolut freiwillig ist und Sie auch keinerlei Nachteile zu erwarten haben, sollten Sie die Teilnahme jetzt beenden.“*

1.5 Studiendesign

Bei der geplanten Arbeit handelt es sich um ein Quasi-Experiment im Rahmen der Grundlagenforschung.

1.6 Studienablauf

Zunächst werden die anthropometrischen Daten, Alter, Geschlecht, Größe und Gewicht von der Versuchsleitung erhoben.

Vor den Messungen werden den Proband*innen Informationen bezüglich der Durchführung der Intervention und Messungen gegeben. Sie nehmen auf einem gepolsterten Stuhl mit Rückenlehne seitlich neben einer Behandlungsliege Platz. Die Sitzposition soll bequem sein und eine Hüftflexion von 120°und eine Knieflexion von 110° implizieren.

Es erfolgt zunächst die Eingangsmessung (Baseline). Anschließend wird für 2 Minuten HILT (OptonPro© 25 Watt – 810 nm, 980 nm, 1064 nm, Zimmer Medizinsysteme GmbH, Neu-Ulm) an der zufällig ausgewählten (rechts oder links, durch Los ermittelt) Patellarsehne appliziert (Abb. 1). Es erfolgt eine postinterventionelle Messung unmittelbar nach der Prozedur, sowie 10 Minuten später.

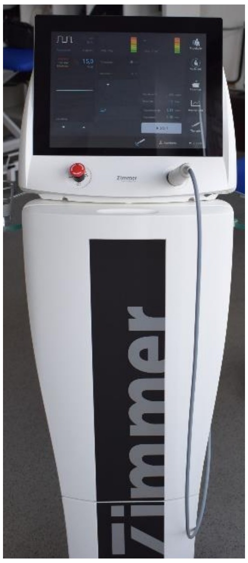

Abbildung 1. OptonPro hochenergetisches Lastertherapiegerät

1.7 Messgrößen

1.7.1 Mikrozirkulation

Die Messung der ligamentären Mikrozirkulation der Patellarsehne erfolgt mittels Laser-Doppler-Flowmeter und Gewebespektrometer (O2C, LEA Medizintechnik GmbH, Heuchelheim) (Abb. 2). Hiermit ist

es möglich in verschiedenen Messtiefen gleichzeitig Blutflussgeschwindigkeit, Blutfluss, Sauerstoffsättigung und relative Hämoglobinmenge des Messgewebes zu bestimmen. Es bestimmt diese Parameter am venösen Ende der Kapillare und gibt somit Auskunft über lokalen Metabolismus, misst also die lokale Mikrozirkulation. Es ist eine Möglichkeit der nichtinvasiven Diagnostik der Mikroperfusion.

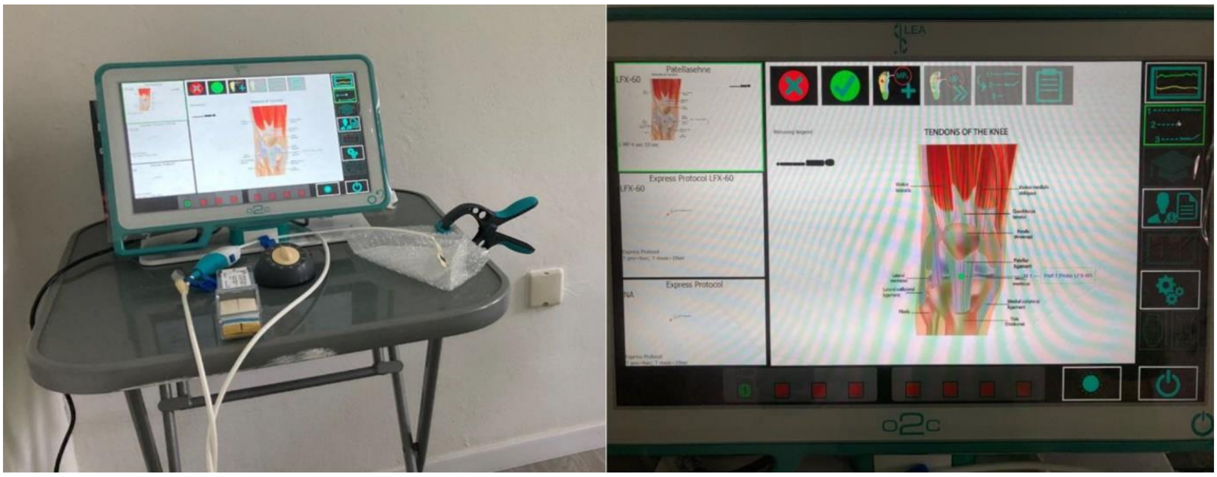

Abbildung 2. Hardware zur Mikrozirkulationsmessung mittels Laser-Doppler-Flowmeter und Gewebespektrometer

1.7.2 Infrarot-Oberflächentemperatur

Hierbei handelt es sich um ein kontaktloses Infrarot-Thermometer (Beurer GmbH, Ulm). Es wird batteriebetrieben und verfügt über eine digitale Anzeige. Das Thermometer ist in der Lage Temperaturen von Oberflächen, Objekten und Flüssigkeiten zu messen. Die Messgenauigkeit bei Objekten/Oberflächen liegt bei +/-3° Celsius (+/- 0,5°F). Der Messbereich liegt bei -22° Celsius bis 80° Celsius.

1.7.3 Thermographie

Bei der Temperaturmessung mittels Thermographie werden Temperaturbereiche von -20° Celsius bis 400° Celsius erfasst (Flir One, Teledyne FLIR LLC, Wilsonville, US). Zur Nutzung der Kamera benötigt es die Hersteller App die kostenfrei zur Verfügung steht. Mittels dieser ist es möglich Bildaufnahmen in Galerien zu ordnen und diese Dateien extern zu speichern. Jede Bilddatei zeigt den aufgenommen Temperaturbereich. Die Messgenauigkeit +/- 3° Celsius oder +/- 5%. Dies gilt 60 Sekunden nach Einschalten des Gerätes in einer Umgebungstemperatur von 15° Celsius bis 35° Celsius und die Zieltemperatur im Bereich von 5° Celsius und 120° Celsius liegt. Ebenso lassen sich mehrere Marker im Bild setzten. Diese Marker lassen eine genaue Gradzahl ablesen. Im angestrebten Experiment werden zwei Marker, einmal mittig der Patellarsehne und ein Areal, das eine Durchschnittstemperatur zeigt, erfasst.

1.8 Fragestellungen

Bisherige Studien zur HILT und deren Wirkung auf die Mikrozirkulation bzw. den Mikrostoffwechsel fehlen oder sind von niedriger Qualität (Kulchitskaya et al., 2016; Angelova et Ilieva, 2016; Wyszyńska et Bal-Bocheńska, 2018). Ziel der Studie ist es daher, einen Beitrag zur Grundlagenforschung auf diesem Gebiet zu leisten. Hierzu werden folgende Forschungsfragen aufgestellt:

- Erzielt eine HILT Behandlung der Patellarsehne signifikante (und/oder klinisch relevante) Temperaturveränderungen in dieser?

- Kann eine HILT Behandlung der Patellarsehne signifikante (und/oder klinisch relevante) Veränderungen der Mikrozirkulation bewirken?

1.9 Störgrößen

Raumtemperatur und Lichtverhältnisse könnten für die Messungen kritische Confounder darstellen. Hierauf ist bei der Studiendurchführung ein besonderes Augenmerk zu richten und für exakt reproduzierbare räumliche Bedingungen Sorge zu tragen.
Eine weitere kritische Variable könnte das Proband*innenalter darstellen, da die Mikrozirkulation von Alterungsprozessen beeinflusst wird. Dieser Einfluss auf die Messung soll durch entsprechende statistische Berücksichtigung vermieden werden.

1.10 Biometrische Auswertung

Es wird angenommen, dass anhand der Stichprobengröße und des Studiendesigns keine Normalverteilung der Variablen vorliegt. Für alle Parameter werden der Median, das erste und dritte Quartil angegeben, sowie eine Signifikanztestung mittels des Wilcoxon-Mann-Whitney-Test als nicht-parametrische Alternative zum Students t-Test durchgeführt.

Für die Datenverwaltung und die Berechnung der deskriptiven Statistik wird das Programm Microsoft Excel 2019 (Microsoft Corporation) genutzt. Für die Berechnung der Inferenzstatistik wird das Statistikprogramm R Version 3.4.1 (Foundation for Statistical Computing) verwendet. Das Signifikanzniveau wird auf p= 0,05 festgelegt.

1.11 Studienorganisation

Es handelt sich um eine monozentrische Studie, die in den Räumen der Diploma Hochschule, Bad Sooden-Allendorf, durchgeführt werden soll. Studienleiter ist Prof. Dr. Christoph Egner, weitere Mitwirkende sind Dr. Robert Schleip, Thomas Gutmann und Andreas Brandl. Die Studie erhält keine externe finanzielle Unterstützung bzw. wird mit eigenen Mitteln durchgeführt.

Als Studienbeginn ist der 10.01.2022 vorgesehen, frühestens jedoch nach Erteilung des Ethikvotums durch die Ethikberatung der Diploma Hochschule und der Registrierung im Deutschen Register Klinischer Studien (DRKS). Die geplante Dauer der Datenerhebung beträgt 12 Wochen.

1.12 Datenschutz

Die geltenden gesetzlichen datenschutzrechtlichen Vorgaben (Datenschutz-Grundverordnung, DSGVO) werden eingehalten. Die Datenerhebung erfolgt anonymisiert. Dies umfasst auch die Speicherung von Bild- und Videomaterial. Die Daten werden nach der Auswertung mittels dem Blockchiffre-Verfahren Advanced Encryption Standard mit einem 128 Bit Schlüssel, passwortgeschützt (mindestens 20 Zeichen, Groß- und Kleinbuchstaben, Sonderzeichen sowie Ziffern) auf einer 25 Gigabyte Blue-Ray Disc gespeichert, von der eine Sicherungskopie angelegt wird. Eine Disc verbleibt in der Diploma Hochschule, das Backup wird in der Praxis Andreas Brandl in einem feuerfesten Tresor untergebracht. Personalisierte Daten werden nicht erhoben und ebenso nicht an dritte weitergegeben.

1.13 Patientensicherheit

Vor Studienbeginn werden alle Teilnehmenden in einem Gespräch über die Inhalte, Ziele und Messverfahren aufgeklärt. Sie werden darüber hinaus ebenso schriftlich informiert und müssen durch ihre Unterschrift die Bereitschaft zur Teilnahme ausdrücken.

Der verwendete Laser ist ein Laser der Klasse 4. Diese Laserstrahlung ist sehr gefährlich für das Auge und gefährlich für die Haut. Auch diffus gestreute Strahlung kann gefährlich sein. Es gilt die Arbeitsschutzverordnung zu künstlicher optischer Strahlung (OStrV). Dementsprechend wird ein

Laserschutzbeauftragter durch den Betreiber benannt, der die Anwender in die Benutzung des Gerätes und die Sicherheit unterweist. Diese verfügen über die rechtlichen Voraussetzungen zur Anwendung medizinischer Laserbehandlung am Menschen . Der Betriebsbereich des Lasers und alle Türen zum Betriebsbereich werden mit Laserwarnschildern versehen. Alle Personen im Behandlungsraum müssen eine Laserschutzbrille mit einer optischen Dichte OD>3 (Schutzstufe mindestens LB 3) bei 810/980/1064 nm für die Betriebsart D (Dauerstrich) und einer Lichtdurchlässigkeit von mindestens 20% im sichtbaren Bereich tragen. Die Brille muss sowohl hitze- als auch UV-beständig sein und die Anforderungen der EN 207 erfüllen. Die Schutzbrille muss für die Leistung von 25 W ausgelegt sein. Die Anwender und Probanden dürfen keine reflektierenden und streuenden Objekte im Behandlungsfeld tragen wie etwa Ringe und Piercings.

Alle Teilnehmenden werden während der Untersuchung ständig betreut und von einer*m erfahrenen und in die Benutzung und Sicherheit des OptonPro eingewiesenen Therapeut*in (>10 Jahre in manueller Therapie, Promotion oder M.Sc. Abschluss) überwacht. Darüber hinaus wird eine Notfallnummer ausgehändigt, sollten außerhalb der Hochschulräume unerwünschte Ereignisse nach der Studienteilnahme auftreten.

Es wird eine Studienregistrierung beim Deutschen Register Klinischer Studien nach dem positiven Ethikvotum der Diploma Hochschule angestrebt. Die Studie stimmt mit der Deklaration von Helsinki in ihrer aktuellen Fassung überein (World Medical Association, 2021).

1.14 Qualitätsmanagement

Die Durchführung der Studie wird entsprechend der geplanten Messabläufe im Sinne standardisierter Verfahrensanweisungen durchgeführt. Die Versuchsleitung wird entsprechend den Anforderungen geschult. Es erfolgt eine Kontrolle qualitätsbezogener Parameter nach jeder Messung eines Teilnehmenden. Dies wird entsprechend protokolliert und dokumentiert. Fehlervorbeugende Maßnahmen im Hinblick auf das Proband*innenhandling und potentiellen Messfehlern wurden erarbeitet und können in der laufenden Studie korrektiv angewandt werden.

2. Informationen für Studienteilnehmende

Proband*inneninformation zur Studie

„Einfluss von hochenergetischer Laserlichtapplikation an der Patellarsehne auf deren ligamentäre Mikrozirkulation. Ein Quasi-Experiment."

Sehr geehrte Probandin, sehr geehrter Proband,

vielen Dank, dass Sie sich bereit erklärt haben, an dieser Studie teilzunehmen. Nachfolgend erhalten Sie hierzu einige Hintergrundinformationen zur Notwendigkeit und zum Zweck dieser Arbeit.

Sinn der Studie

Nicht-invasive Therapiegeräte erlangen im Rahmen der Behandlung und Rehabilitation von Erkrankungen des Muskel- und Skelettapparates eine immer wichtigere Bedeutung. Die hochenergetische Lasertherapie ist hierbei eine relativ neue Methode. Bisher ist jedoch wenig über die Wirkmechanismen hinter dieser Therapieform bekannt. Diese Zusammenhänge näher zu untersuchen ist von besonderem wissenschaftlichem Interesse. In dieser Studie sollen hierzu an Gesunden Menschen Messungen durchgeführt und Daten erhoben werden, um Erkenntnisse zu gewinnen, wie sich die Lasertherapie an der Sehne unterhalb der Kniescheibe auf die Durchblutung auswirkt.

Studienablauf

Es wird mit Ihnen ein Termin (ca. 45 Minuten) vereinbart. Nach einem Informationsgespräch werden die Temperatur und Durchblutung Ihrer Sehne unterhalb der Kniescheibe gemessen. Dies erfolgt auf der Haut und ist schmerzfrei. Im Anschluss wird mittels eines hochenergetischen Laserlichts die Sehne behandelt. Während dieser Behandlung müssen Sie eine Schutzbrille tragen, die Ihre Augen vor Laserstrahlen abschirmt. Sie dürfen keine reflektierenden oder streuende Objekte wie Ringe oder Piercings im Behandlungsgebiet tragen. Auch diese Behandlung ist berührungslos und im Allgemeinen schmerzfrei. Direkt anschließend und 10 Minuten später wird nochmals Temperatur und Durchblutung der Sehne gemessen.

Die Covid 19 Pandemie erfordert in dieser Studie einen besonderen Schutz der Probanden und Probandinnen, daher werden während der gesamten Studie die aktuellen Hygienerichtlinien eingehalten.

Mögliche Risiken

Die Laser-Therapie hat bei korrekter Anwendung keine Nebenwirkungen. Während der Laserlicht-Behandlung kann es jedoch in manchen Fällen zu unangenehmen Temperaturempfinden und selten auch in deren Verlauf zu einem Schmerz durch punktuelle Wärme kommen. Hier besteht immer die Möglichkeit die Behandlung abzubrechen, das Laserlicht wird dann sofort abgeschaltet, sodass eine weitere Schmerzwirkung abrupt unterbunden wird.

Persönlicher Nutzen der Studie

Mit Ihrer Teilnahme an der Studie leisten Sie einen wertvollen Beitrag zur humanwissenschaftlichen Forschung. Mit Ihrer Hilfe können Grundlagendaten über die Wirkung der Laserlicht-Therapie gesammelt werden, um in der Zukunft Bindegewebs- und Muskelerkrankungen effektiver behandeln zu können. Ein unmittelbarer Vorteil entsteht Ihnen nicht.

Teilnahmekriterien

Sie müssen zwischen 18 und 50 Jahren alt sein und über eine allgemein gesunde Konstitution verfügen. Nicht teilnehmen können Sie leider, wenn Sie an psychischen Vorerkrankungen, akuten Entzündungen oder großflächigen Narben am Kniegelenk leiden. Sie sollten keine Operationen in den letzten drei Monaten, keine Kniegelenksprothese, wissentliche Verwachsungen oder Hämatome in diesem Bereich haben.

Zur Abklärung dieser Teilnahmebedingungen steht Ihnen eine Checkliste zu Ihrem persönlichen Gebrauch zur Verfügung.

Kosten

Für die Teilnahme an der Studie entstehen Ihnen natürlich keine Kosten.

Freiwilligkeit der Teilnahme und Rücktrittsrecht

Die Teilnahme an der Studie ist freiwillig. Sie können jederzeit und ohne Angabe von Gründen die Teilnahme an dieser Studie beenden, ohne dass Ihnen daraus Nachteile entstehen. Auch wenn Sie die Studie vorzeitig abbrechen, haben Sie keine Nachteile.

Bestätigung der Vertraulichkeit und Datenschutz

Die im Rahmen der Studie nach Einverständniserklärung erhobenen persönlichen Daten unterliegen der Schweigepflicht und den datenschutzgesetzlichen Bestimmungen. Sie werden in Papierform aufgezeichnet und in Obhut der Diploma Hochschule verwahrt. Die Nutzung der Daten erfolgt in verschlüsselter Form, d.h. die ermittelten Daten werden nicht unter Ihrem Namen gespeichert, sondern unter einem anonymen Zahlencode. Eine Weitergabe der erhobenen Daten im Rahmen des Forschungszwecks erfolgt nur in verschlüsselter Form. Gleiches gilt für die Veröffentlichung der Studienergebnisse.

Die Aufzeichnung bzw. Speicherung erfolgt für die Dauer von 10 Jahren.

3. Einwilligungserklärung

Einverständniserklärung der Patientin / des Patienten zur Studie

„Einfluss von hochenergetischer Laserlichtapplikation an der Patellarsehne auf deren ligamentäre Mikrozirkulation. Ein Quasi-Experiment.“

Name: ____________________
Vorname: ____________________
Geburtsdatum: ____________________

Ich, ______________________________, wurde von der Versuchsleitung, __________________________ , über Wesen, Bedeutung und Tragweite der oben genannten Studie aufgeklärt. Ich habe den Aufklärungstext gelesen und verstanden. Ich hatte die Möglichkeit, Fragen zu stellen und habe die Antworten verstanden und akzeptiere sie. Die Versuchsleitung hat mich über die mit der Teilnahme an der Studie verbundenen Risiken und den möglichen Nutzen informiert.

Ich hatte ausreichend Zeit, mich zur Teilnahme an dieser Studie zu entscheiden und weiß, dass die Teilnahme an dieser Studie freiwillig ist. Ich weiß, dass ich jederzeit und ohne Angaben von Gründen diese Zustimmung widerrufen kann, ohne dass sich dieser Entschluss nachteilig auf die spätere Behandlung durch meinen Therapeuten auswirken wird.

Mir ist bekannt, dass diese Einverständniserklärung in die Obhut der Diploma Hochschule übergeht und von dieser nach den datenschutzgesetzlichen Bestimmungen verwahrt wird.

Checkliste zur Studienteilnahme

Wissenschaftliche Experimente und Studien müssen unter nachvollziehbaren Bedingungen mit einer möglichst einheitlichen Gruppe an Studienteilnehmenden durchgeführt werden. Sollten Sie eine der nachfolgenden Fragen mit **„ja“** beantworten, können Sie leider nicht an der Studie teilnehmen, was natürlich kein persönliches Manko darstellt, sondern den Anforderungen an quasi Laborbedingungen geschuldet ist.

☐ **ja** ☐ **nein** Sie haben das 18. Lebensjahr noch nicht oder das 49. Lebensjahr bereits beendet.

☐ **ja** ☐ **nein** Sie hatten eine Operation in den letzten drei Monaten.

☐ **ja** ☐ **nein** Sie wissen von Verwachsungen, Hämatomen oder Hauterkrankungen im Bereich des Kniegelenks (Kniegelenk und jeweils 10 cm des angrenzenden Ober- oder Unterschenkels).

☐ **ja** ☐ **nein** Sie leiden unter einer akuten oder chronischen Entzündung des Kniegelenks.

☐ **ja** ☐ **nein** Sie waren in den letzten zwei Jahren in psychotherapeutischer und/oder in psychiatrischer Behandlung.

☐ **ja** ☐ **nein** Sie haben größere Narben (2 cm Länge und größer) im Bereich des Kniegelenks (Kniegelenk und jeweils 10 cm des angrenzenden Ober- oder Unterschenkels).

☐ **ja** ☐ **nein** Bei Ihnen wurde eine Kniegelenksprothese eingesetzt.

☐ **ja** ☐ **nein** **Sie verfügen über kein intaktes thermisches Empfinden und können Schmerzen nicht oder vermindert wahrnehmen und kommunizieren.**

☐ **ja** ☐ **nein** **Sie nehmen Schmerzmittel oder bewusstseinsverändernde Substanzen ein.**

Mit meinem Einverständnis zur Teilnahme erkläre ich, die Checkliste zur Studienteilnahme nach bestem Wissen beantwortet zu haben und dass ich mit der im Rahmen dieser Studie erfolgenden anonymen Aufzeichnung von Untersuchungs- und Krankheitsdaten einverstanden bin.
Ich habe eine Kopie der Studieninformationen und dieser Einwilligungserklärung erhalten.
Ich erkläre hiermit meine freiwillige Teilnahme an dieser Studie.

___________________________ ___________________________

Ort und Datum — Unterschrift der Patientin / des Patienten

___________________________ ___________________________

Ort und Datum — Unterschrift der aufklärenden Versuchsleitung

5 Literatur

Angelova, A., Ilieva, E. M. (2016). *Effectiveness of High Intensity Laser Therapy for Reduction of Pain in Knee Osteoarthritis*. Pain Research and Management 2016, 1–11.

Hamblin, M., 1 Wellman Center for Photomedicine, Massachusetts General Hospital, BAR414, 40 Blossom Street, Boston, MA 02114, USA, 2 Department of Dermatology, Harvard Medical School, Boston, MA 02115, USA, 3 Harvard-MIT Division of Health Sciences and Technology, Cambridge, MA 02139, USA (2017). *Mechanisms and Applications of the Anti-Inflammatory Effects of Photobiomodulation*. AIMS Biophysics 4 (3), 337–361.

Kulchitskaya, D. B., Konchugova, T. V., Fedorova, N. E. (2016). *Comparative evaluation of the effects of high- intensity and low-intensity laser radiation on microcirculation among patients with knee arthritis*. Journal of Physics: Conference Series 755, 011001.

Mussttaf, R. A., Jenkins, D. F. L., Jha, A. N. (2019). *Assessing the Impact of Low Level Laser Therapy (LLLT) on Biological Systems: A Review*. International Journal of Radiation Biology 95 (2), 120–143.

Spieß, C. (2021). *Untersuchung des Einflusses von induzierten Temperaturwechseln auf die Durchblutung der Patellar-Sehne*. Diploma Hochschule.

Tkocz, P., Matusz, T., Kosowski, Ł., Walewicz, K., Argier, Ł., Kuszewski, M., Hagner-Derengowska, M., Ptaszkowski, K., Dymarek, R., Taradaj, J. (2021). *A Randomised-Controlled Clinical Study Examining the Effect of High-Intensity Laser Therapy (HILT) on the Management of Painful Calcaneal Spur with Plantar Fasciitis*. Journal of Clinical Medicine 10 (21), 4891.

Tumilty, S., Munn, J., McDonough, S., Hurley, D. A., Basford, J. R., Baxter, G. D. (2010). *Low Level Laser Treatment of Tendinopathy: A Systematic Review with Meta-Analysis*. Photomedicine and Laser Surgery 28 (1), 3–16.

Willard, F. H., Vleeming, A., Schuenke, M. D., Danneels, L., Schleip, R. (2012). *The Thoracolumbar Fascia: Anatomy, Function and Clinical Considerations*. Journal of Anatomy 221 (6), 507–536.

Wyszyńska, J., Bal-Bocheńska, M. (2018). *Efficacy of High-Intensity Laser Therapy in Treating Knee Osteoarthritis: A First Systematic Review*. Photomedicine and Laser Surgery 36 (7), 343–353.
